# Long-term response after electrochemotherapy in patients with relapsed or refractory cutaneous melanoma

**DOI:** 10.1186/1479-5876-12-S1-P1

**Published:** 2014-05-06

**Authors:** Nicola Mozzillo, Corrado Caracò, Ugo Marone, Ester Simeone, Stefano Mori, Lucia Benedetto, Gianluca Di Monta, Maria Luisa Di Cecilia, Massimiliano Di Marzo, Gerardo Botti, Paolo Antonio Ascierto

**Affiliations:** 1 Unit of Surgery “Melanoma - Soft Tissues”, National Cancer Institute, Naples, Italy; 2 Unit of Medical Oncology, National Cancer Institute, Naples, Italy; 3 Unit of Pathology, National Cancer Institute, Naples, Italy

## Background

Treatment of early and multiple cutaneous unresectable recurrences is a major therapeutic problem with around 80% of patients relapsing within 5 years [1]. For lesions refractory to elective treatments, electrochemotherapy (ECT) involving electroporation combined with antineoplastic drug treatment appears to be a new potential option [2]. This study was undertaken to analyze the short- and long-term responses of lesions treated with ECT with intravenous injection of bleomycin in melanoma patients with in-transit disease or distant cutaneous metastases.

## Materials and methods

Between January 2007 and September 2012, 60 patients with relapsed and refractory cutaneous melanoma metastases or in-transit disease underwent 100 courses of ECT with intravenous injection of bleomycin. Response to treatment was evaluated three months after ECT. A long-lasting response was defined as no cutaneous or in-transit relapse after a minimum of six months.

## Results

Three months after ECT, a complete response was observed in 29 patients (48.4%), a partial response in 23 patients (38.3%) and no change or progressive disease in 8 patients (13.3%). The objective response rate of all treated lesions was 86.6%. Thirteen patients (44.8% of complete responders) experienced a long-lasting response to ECT and were disease-free after a mean duration of follow-up of 27.5 months.

## Conclusions

The favorable outcome obtained in the present study demonstrates that ECT is a reliable, and effective procedure that provides long-term benefit in terms of curative and palliative treatment for unresectable cutaneous lesions without adversely impacting the quality of life of patients [3-7].
